# Individual and environmental determinants associated with longer times to access pediatric rheumatology centers for patients with juvenile idiopathic arthritis, a JIR cohort study

**DOI:** 10.1186/s12969-023-00809-8

**Published:** 2023-03-14

**Authors:** Aurélie Chausset, Céline Lambert, Alexandre Belot, Etienne Merlin, Elvira Cannizzaro, Isabelle Kone-Paut, Claire Ballot, Valérie Devauchelle, Sylvaine Poignant, Raffaella Carlomagno, Anne Lohse, Catherine Barbier, Véronique Despert, Aurélia Carbasse, Laetitia Sparsa, Eva Adank, Federica Vanoni, Héloise Reumaux, Pascal Pillet, Daniela Kaiser, Michael Hofer, Caroline Freychet, Anne-Marie Schott

**Affiliations:** 1 CRECHE Unit, INSERM CIC 1405, Department of Pediatrics, CHU Clermont-Ferrand, Clermont-Ferrand, France; 2 Research on Healthcare Performance (RESHAPE), INSERM U1290, Claude Bernard University Lyon 1, Lyon, France; 3 Pédiatrie, CHU Estaing, 1 Place Lucie & Raymond Aubrac, Clermont-Fd cedex1, 63003 France; 4 Biostatistics Unit, DRCI, CHU Clermont-Ferrand, Clermont-Ferrand, France; 5 Pediatric Nephrology, Rheumatology, Dermatology, HFME, Hospices Civils de Lyon, National Referee Centre RAISE & INSERM U1111, Lyon University, Lyon, France; 6 Department of Rheumatology, University Children’s Hospital, University of Zurich, Zurich, Switzerland; 7 Department of Pediatrics & Pediatric Rheumatology, Centre de référence maladies auto-inflammatoires rares et amylose inflammatoire (CEREMAIA), Bicêtre University, Paris Sud Hospital, Le Kremlin-Bicêtre, France; 8 Pediatric Hematology, Jean-Minjoz Hospital, Besançon, France; 9 Rheumatology Department, CHU Brest and Brest University, INSERM UMR 1227, Brest, France; 10 General Pediatrics, CHU Nantes, Nantes, France; 11 Pediatric Immuno-Rheumatology of Western Switzerland, Department Women-Mother-Child, Lausanne University Hospital, Lausanne, Switzerland; 12 University Hospital, Geneva, Switzerland; 13 Department of Rheumatology, Nord Franche-Comté Hospital, Belfort, France; 14 Pediatric Immunology, Albert Michallon Hospital, Grenoble, France; 15 Department of Pediatrics, CHU Rennes, Rennes, France; 16 Department of Pediatrics, Hôpital Arnaud de Villeneuve, CHRU Montpellier, Montpellier, France; 17 Rheumatology, CH Mulhouse, Mulhouse, France; 18 Department of Pediatrics, Kantonsspital Graubünden, Chur, Switzerland; 19 Institute of Pediatrics of Southern Switzerland, EOC, Bellinzona, Switzerland; 20 Pediatric Rheumatology, Lille University Hospital, Lille, France; 21 Department of Pediatrics, Hôpital des Enfants, CHRU Bordeaux, Bordeaux, France; 22 Department of Pediatrics, Centre Hospitalier Cantonal Luzern, Lucerne, Switzerland; 23 Pediatrics, CHU Saint-Etienne, Saint-Etienne, France

**Keywords:** Juvenile idiopathic arthritis, Access to care, Time to referral, Socio-economic factors

## Abstract

**Background:**

Despite guidelines, poor access to appropriate care for juvenile idiopathic arthritis (JIA) patients remains a global issue. Prompt referral to a pediatric rheumatology (PR) center and effective care is known to be critical for changing the natural history of the disease and improving long-term prognosis. This project assesses socio-economic factors of delayed referral to a pediatric rheumatologist (PRst) for JIA patients in France and Switzerland within the Juvenile Inflammatory Rheumatism (JIR) Cohort.

**Methods:**

All patients diagnosed with JIA, presenting at one center of the JIRcohort in France or Switzerland with additional data on referral pathway were included. Patient characteristics at first visit to the PR center, dates of visits to healthcare providers during referral, and parent characteristics were extracted from the JIRcohort database.

**Results:**

Two hundred fifty children were included. The overall median time to first PR assessment was 2.4 months [1.3; 6.9] and ranged widely across the JIA subtypes, from 1.4 months [0.6; 3.8] for children with systemic juvenile idiopathic arthritis (sJIA) to 5.3 months [2.0; 19.1] for children with enthesitis-related arthritis (ERA). A diagnosis of ERA and an appointment with an orthopedist during the referral pathway were significantly associated with a longer time before the first PR visit (hazard ratio HR 0.50 [95% CI: 0.29; 0.84]) and HR 0.68 [95% CI: 0.49; 0.93], respectively) in multivariable analysis. Having a mother with a post-graduate educational attainment level was tendentially associated with a shorter time before the first PR visit, (HR 1.32 [95% CI: 0.99; 1.78]).

**Conclusions:**

Time to first PRst visit was most often short compared to other studies and close to the British recommendations. However, this time remained too long for many patients. We observed no social inequities in access to a PRst, but we show the need to improve effective pathway and access to a PR center for JIA patients.

**Supplementary Information:**

The online version contains supplementary material available at 10.1186/s12969-023-00809-8.

## Background

Juvenile idiopathic arthritis (JIA) is the most common chronic pediatric rheumatic disease [1]. It is defined by the onset, before age 16 years, of arthritis of unknown cause persisting for at least 6 weeks [2]. The term JIA encompasses a heterogeneous group of different diseases classified into seven categories of varying severity and long-term consequences depending on clinical manifestations and response to treatment. JIA qualifies as a rare disease (prevalence less than 1/2000 children) but is widely underdiagnosed [3].

Prompt referral to a pediatric rheumatology (PR) center and effective care is known to be critical in changing the natural history of the disease and improving long-term prognosis [4–11]. Delay in diagnosis can also be a source of anxiety, or of non-adherence to treatment, especially in case of loss of confidence in healthcare providers (HCPs). The care pathway for JIA patients can be complex, and reasons for delayed referral may depend on several factors such as individual patient characteristics and local and regional healthcare organization. JIA can also be under-recognized by HCPs because of its low prevalence and subtle clinical manifestations.

International guidelines advocate that whatever the level of income of the country, new patients with suspected JIA should be assessed by a pediatric rheumatologist (PRst) within 4 weeks from the time of referral [12]. In addition, the British Society for Paediatric and Adolescent Rheumatology Standards of Care (BSPAR) and the Arthritis and Musculoskeletal Alliance advocate that children with suspected JIA should be assessed by a PR team within 10 weeks of symptom onset [13].

Despite guidelines, poor access for JIA patients to appropriate care remains a global issue. Literature reports give a median time to access the PRst of 3–10 months, with many medical stakeholders involved [14–26] and a broad variability in JIA subtypes [26]. It was found that some clinical characteristics and biological factors such as joint swelling, fever, and elevated C-reactive protein/erythrocyte sedimentation rate were associated with a shorter time to first PR visit. Conversely, enthesitis, older age at symptom onset/diagnosis or pain were associated with a longer time to access PR centers [15, 16, 20, 27].

Data on the impact of socio-economic status on time to access to PR center are scant, and available only in North America and the United Kingdom [15, 22, 28]. Because health in childhood is influenced by socio-economic determinants [29, 30], we sought to identify potential socio-economic determinants of delayed referral to a PRst for JIA patients in a cohort set up in France and Switzerland.

## Methods

### Study design

The Juvenile Inflammatory Rheumatism cohort (JIRcohort) is an international multicenter prospective data repository where patients with juvenile inflammatory rheumatisms are collected in a web-secured database (clinicaltrial: NCT02377245) [31].

The study was conducted in accordance with the Declaration of Helsinki and the protocol was approved by independent ethics committees for each participating center.

### Definitions and variables

Time to first PR visit was defined as the time from the onset of symptoms to the first visit to a PR center.

Time to first HCP visit was defined as the time between symptom onset and first assessment by an HCP.

Time between first HCP and first PR visit was defined as the time between first consultation with an HCP and first PR assessment.

HCP specialties recorded were pediatric rheumatologist (PRst), general pediatrician, general practitioner, emergency care practitioner (ECP), orthopedist, and other.

The distance from parents’ dwelling place to the PR center was calculated using an Internet-based route calculator (URL: https://fr.mappy.com) as the shortest distance to the PR center by road.

Patients’ living area were classified as rural, intermediate (i.e., both rural and urban parts or rural under strong influence of an urban area) or urban (according to INSEE, the French National Statistical Office [32], and the Swiss Federal Statistical office [33]).

Parental profession was recorded using the International Standard Classification of Occupations (ISCO) [34]. Parental educational attainment was recorded using the International Standard Classification of Education (ISCED) [35].

To assess the impact of closeness to medical and/or health systems on time to access a PR center. We separated parental professions into two categories: parents with health care profession (e.g., physician, nurse, laboratory technician, pharmacist, or physiotherapist) and others. By using parental occupation as a proxy, we aimed to study whether having parents in the medical field had an impact on access to care as suggested in other studies [36, 37].

### Population

All patients diagnosed with JIA (according to the International League of Associations for Rheumatology classification [38]), presenting at one center of the JIRcohort in France or in Switzerland were included (HCPs met during referral and dates of visits).

The overall cohort was started in 2013. The data analyzed here were for a subcohort for which socio-economic data (which were not initially collected) had been collected from January 2018 to April 2019. The patients included were those who had been managed since the data were collected and those being followed in the PR center (and for whom data could be completed).

### Data collection

Data was collected by a PRst in each center during a follow-up visit.

Data on patients’ characteristics at first visit to PR center (age, JIA subtype) and parents’ characteristics (profession and educational attainment) were extracted from the JIRcohort database. The referral pathway to PR center was also described, i.e., the specialty of each HCP met by the patient for JIA related symptoms, and the timing (the date of first medical appointment with this specialist). If parents had forgotten the exact date, and if only the month and year were available, an approximation to within 15 days was recorded.

### Analysis

Statistical analysis was performed using the Stata software (version 15; StataCorp, College Station, Texas, USA). All tests were two-sided, with an alpha level set at 0.05. Categorical data were expressed as number of subjects and associated percentages, and continuous data as median [25th; 75th percentile]. The primary outcome was estimated using the Kaplan–Meier approach, and factors associated with time to first PR visit were studied using the log-rank statistic in univariate analysis. A multivariable analysis was then performed using a Cox proportional hazards model, considering covariates determined according to univariate results and clinical relevance. The results were expressed as hazard ratios (HR) and 95% confidence interval (CI). An HR of 1 indicates an equal likelihood of first PR visit in the presence of the variable in question as in its absence, HR > 1 indicates an increased likelihood (shorter time), and HR < 1 a reduced likelihood (longer time). A logarithmic transformation of the distance from the patient’s dwelling place to the PR center was carried out to achieve normality.

Factors associated with the time between symptom onset and first consultation with an HCP and the time between first consultation with an HCP and first PR assessment were also studied using the log-rank statistic.

## Results

Of the 1342 JIA patients enrolled in the initial JIRcohort database, socio-economic factors and referral pathways were collected in 250 children (41 in Switzerland, 209 in France), in 20 centers (Additional file 1).

### Characteristics at first visit to PR centers

Characteristics of JIA patients and parents at first visit to a PR center are reported in Table 1.

**Table 1:** Characteristics of juvenile idiopathic arthritis patients and parents at first visit to a pediatric rheumatology center

	**Whole sample (*n* = 250)**
**Patient characteristics**	
Age at diagnosis (years)	4.8 [2.5; 9.4]
Age at onset (years)	4.3 [2.1; 8.4]
Female sex	190 (76.0%)
Distance from patient’s dwelling place to the PR center (km)	37 [17; 82]
**Patient location area**	
Rural	75/250 (30.0%)
Intermediate	41/250 (16.4%)
Urban	134/250 (53.6%)
**Mother’s educational level**	
Middle school (lower secondary in Switzerland)	16/230 (7.0%)
High school (upper secondary in Switzerland)	58/230 (25.0%)
Post-baccalaureate studies < 3 years (short cycle higher education)	54/230 (23.5%)
3-year post-baccalaureate studies (bachelor’s degree or equivalent)	47/230 (20.5%)
5-year post-baccalaureate studies (master’s degree or equivalent)	39/230 (17.0%)
> 5 years (doctorate or equivalent)	16/230 (7.0%)
**Father’s educational level**	
Middle school (lower secondary in Switzerland)	22/220 (10.0%)
High school (upper secondary in Switzerland)	60/220 (27.3%)
Post-baccalaureate studies < 3 years (short cycle higher education)	53/220 (24.1%)
3-year post-baccalaureate studies (bachelor’s degree or equivalent)	30/220 (13.6%)
5-year post-baccalaureate studies (master’s degree or equivalent)	42/220 (19.1%)
> 5 years (doctorate or equivalent)	13/220 (5.9%)
**Parents’ occupation**	
Mother in a healthcare profession	28/240 (11.7%)
Father in a healthcare profession	8/226 (3.5%)
At least one parent in a healthcare profession	31/227 (13.7%)

Data are number of subjects (associated percentages) or median [25th; 75th percentile]

*PR* Pediatric rheumatology

The median age at onset was 4.3 years [2.1; 8.4] and 76% of the children were female.

The median distance from the patient’s dwelling place to the PR center was 37 km [17; 82] with a maximum of 407 km and more than half of the patients lived in an urban area (134/250).

Regarding educational attainment level of parents, 68% of mothers and 63% of fathers had a post-graduate level (university degree or equivalent).

Fourteen percent of children had at least one parent working in a medical or paramedical profession (12% mothers and 4% fathers).

### Time to referral

Time to access the PR center is reported in Table 2 and broken down into JIA subtypes. The most frequent JIA subtype was oligoarticular (oJIA) (50%), then polyarticular (pJIA) (22%), then enthesitis-related arthritis (ERA) (11%).

**Table 2:** Characteristics and time to access pediatric rheumatology center by juvenile idiopathic arthritis subtype

	**Whole sample (*n* = 250)**	**oJIA (*n* = 124)**	**pJIA (*n* = 56)**	**ERA (*n* = 27)**	**sJIA (*n* = 23)**	**PsoJIA (*n* = 11)**	**UndJIA (*n* = 9)**
Age at diagnosis (years)	4.8 [2.5; 9.4]	3.3 [2.1; 5.2]	5.3 [2.9; 9.2]	11.1 [8.5; 12.4]	6.2 [3.2; 10.6]	10.2 [6.8; 11.5]	12.7 [11.8; 14.4]
Age at onset (years)	4.3 [2.1; 8.4]	2.7 [1.7; 4.8]	4.9 [2.5; 8.8]	9.1 [6.7; 11.7]	5.4 [2.8; 10.3]	9.1 [6.2; 10.9]	12.2 [9.9; 13.5]
Female sex	190 (76.0%)	104 (83.9%)	43 (76.8%)	12 (44.4%)	17 (73.9%)	7 (63.6%)	7 (77.8%)
Time between onset and first HCP (months)	0.0 [0.0; 0.7]	0.0 [0.0; 0.4]	0.1 [0.0; 0.7]	0.8 [0.0; 5.0]	0.0 [0.0; 0.3]	0.0 [0.0; 0.5]	0.0 [0.0; 0.5]
Time between first HCP and PR visit (months)	2.1 [1.0; 5.0]	2.1 [0.9; 4.6]	2.4 [1.2; 5.4]	4.0 [1.7; 8.0]	1.1 [0.3; 1.6]	2.1 [0.7; 9.0]	2.3 [1.2; 4.1]
Time to first PR visit (months)	2.4 [1.3; 6.9]	2.2 [1.3; 5.0]	3.0 [1.5; 6.9]	5.3 [2.0; 19.1]	1.4 [0.6; 3.8]	2.7 [1.2; 12.9]	2.9 [1.2; 6.0]
Time to first PR visit ≥ 10 weeks	132 (52.8%)	60 (48.4%)	33 (58.9%)	20 (74.1%)	7 (30.4%)	6 (54.5%)	6 (66.7%)
Time to first PR visit ≥ 6 months	67 (26.8%)	29 (23.4%)	15 (26.8%)	13 (48.1%)	4 (17.4%)	4 (36.4%)	2 (22.2%)
Time to first PR visit ≥ 12 months	36 (14.4%)	15 (12.1%)	5 (8.9%)	9 (33.3%)	2 (8.7%)	3 (27.3%)	2 (22.2%)
Number of HCPs met before the PRst	2 [1; 3]	2 [1; 3]	2 [2; 3]	2 [1; 3]	2 [2; 3]	2 [1; 2]	2 [1; 3]
Distance from patient’s dwelling place to the PR center (km)	37 [17; 82]	41 [15; 95]	37 [24; 61]	40 [19; 125]	45 [14; 70]	30 [15; 43]	23 [19; 31]

Data are number of subjects (associated percentages) or median [25th; 75th percentile]

*oJIA* Oligoarticular juvenile idiopathic arthritis, *pJIA* Polyarticular juvenile idiopathic arthritis, *ERA* Enthesitis-related arthritis, *sJIA* Systemic juvenile idiopathic arthritis, *PsoJIA* Psoriatic arthritis, *UndJIA* Undifferentiated juvenile idiopathic arthritis, *HCP* Healthcare provider, *PR* Pediatric rheumatology, *PRst* Pediatric rheumatologist

The overall median time between onset and first PR assessment was 2.4 months [1.3; 6.9] and varied considerably across the JIA subtypes, from 1.4 months [0.6; 3.8] for children with sJIA to 5.3 months [2.0;19.1] for children with ERA.

More precisely, the median time between first symptoms and first visit to an HCP was very short (0.0 month [0.0; 0.7]), whereas the median time between this first consultation with an HCP and first PR visit was 2.1 months [1.0; 5.0].

Only 47% of children were assessed by a PRst within 10 weeks after onset of symptoms (BSPAR guidelines) and about one quarter of the patients (27%) were seen by a PRst 6 months or more after first symptoms. Among ERA patients, time to PR visit was more than 6 months for approximately half (48%) and more than 12 months for one third (33%).

### Factors associated with delay in access to PR centers

Based on univariate analysis, an appointment with an orthopedist during the referral pathway and a diagnosis of ERA were significantly associated with a longer time before the first PR visit (HR 0.71 [95% CI: 0.53; 0.94]) and (HR 0.47 [95% CI 0.30; 0.73], respectively) (Table 3). By contrast, patients with an appointment with an ECP and a mother with a post-graduate educational level were more likely to experience a shorter time before the first PR visit (HR 1.36 [95% CI: 1.06; 1.75] and HR 1.38 [95% CI 1.04; 1.83], respectively).

**Table 3:** Predictive factors of time to first pediatric rheumatology visit

	**Univariate**		
	**HR**	**95% CI**	***p***
Female sex	1.25	0.93; 1.68	0.13
Age at onset	0.98	0.95; 1.01	0.19
Distance from patient’s dwelling place to the PR center^a^	0.92	0.82; 1.02	0.11
JIA subtype			
oJIA	Ref		
pJIA	0.91	0.66; 1.25	0.56
ERA	0.47	0.30; 0.73	0.001
sJIA	1.25	0.80; 1.97	0.33
PsoJIA	0.79	0.42; 1.46	0.45
UndJIA	0.75	0.38; 1.48	0.40
Country			
France	Ref		
Switzerland	0.96	0.68; 1.34	0.79
Patient location area			
Urban	Ref		
Intermediate	0.93	0.65; 1.32	0.69
Rural	0.96	0.72; 1.27	0.77
General pediatrician met during referral	1.03	0.80; 1.33	0.80
Emergency care practitioner met during referral	1.36	1.06; 1.75	0.016
Orthopedist met during referral	0.71	0.53; 0.94	0.019
Mother with post-graduate level	1.38	1.04; 1.83	0.025
Father with post-graduate level	1.14	0.86; 1.51	0.36
At least one parent in a health care profession	1.18	0.80; 1.73	0.40

HR = 1 indicates an equal likelihood of a short time to first PR visit in the presence of the variable in question as in its absence. HR > 1 indicates an increased likelihood of a short time to first PR visit. HR < 1 indicates a reduced likelihood of a short time to PR visit

*HR* Hazard ratio, *Ref* Reference, *CI* Confidence interval, *PR* Pediatric rheumatology, *oJIA* Oligoarticular juvenile idiopathic arthritis, *pJIA* Polyarticular juvenile idiopathic arthritis, *sJIA* Systemic juvenile idiopathic arthritis, *ERA* Enthesitis-related arthritis, *PsoJIA* Psoriatic arthritis, *UndJIA* Undifferentiated juvenile idiopathic arthritis

^a^With a logarithmic transformation

Country and distance to PR center were not associated with time to access a PRst (respectively HR 0.96 [95% CI: 0.68; 1.34] and HR 0.92 [95% CI: 0.82; 1.02]).

**Fig. 1:**
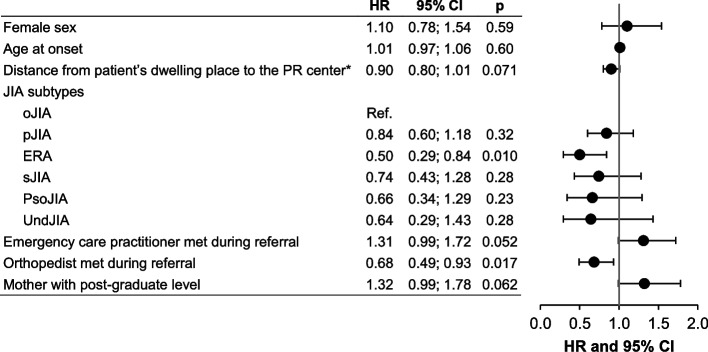
Multivariable analysis of predictive factors for time to first pediatric rheumatologist visit (*n* = 230). HR = 1 indicates an equal likelihood of a short time to first PR visit in the presence of the variable in question as in its absence. HR > 1 indicates an increased likelihood of a short time to first PR visit. HR < 1 indicates a reduced likelihood of a short time to PR visit. JIA: juvenile idiopathic arthritis; oJIA: oligoarticular JIA; pJIA: polyarticular JIA; sJIA: systemic JIA; ERA: enthesitis-related arthritis; PsoA: psoriatic arthritis; UndJIA: undifferentiated JIA; HR: hazard ratio; CI: confidence interval; Ref: reference. * With a logarithmic transformation

In multivariable analysis, ERA subtype (HR 0.50 [95% CI: 0.29; 0.84]) and appointment with an orthopedist (HR 0.68 [95% CI: 0.49; 0.93]) remained independent factors associated with longer time to access a PRst, whereas visit to an ECP was almost significantly associated with a shorter delay (HR 1.31 [95% CI: 0.99; 1.72]) (Fig. 1). Similarly, having a mother with a post-graduate educational level was tendentially associated with a shorter time before the first PR visit (HR 1.32 [95% CI: 0.99; 1.78]).

Based on univariate analysis, having a mother with post-graduate level, and living in rural area were significantly associated with a shorter time between symptom onset and first visit to an HCP (HR 1.36 [95% CI:1.02; 1.81] and HR 1.34 [95% CI:1.00; 1.78], respectively). Conversely, a diagnosis of ERA was significantly associated with a longer time (HR 0.45 [95% CI:0.28; 0.70]) (Table 4). Regarding the time between first consultation with an HCP and first PRst assessment, a longer distance from patient’s dwelling place to the PR center was associated with a longer time, while a diagnosis of sJIA was associated with a shorter time (HR 0.84 [95% CI 0.75; 0.93] and HR 1.84 [95% CI 1.17; 2.87], respectively) (Table 4).

**Table 4:** Predictive factors of time of the two periods of the referral (time between symptom onset and first visit with an HCP and time between first visit with an HCP and first PR assessment)

	**Time between onset and first HCP (months)**			**Time between first HCP and first PR visit (months)**		
	**HR**	**95% CI**	***p***	**HR**	**95% CI**	***p***
Female sex	1.27	0.95; 1.71	0.11	0.99	0.74; 1.33	0.95
Age at onset	0.98	0.95; 1.02	0.35	0.98	0.95; 1.02	0.30
Distance from patient’s dwelling place to the PR center^a^	1.06	0.95; 1.18	0.32	0.84	0.75; 0.93	0.001
JIA subtype						
oJIA	Ref			Ref		
pJIA	0.92	0.67; 1.26	0.61	0.91	0.66; 1.26	0.58
ERA	0.45	0.28; 0.70	0.001	0.72	0.47; 1.11	0.13
sJIA	0.89	0.57; 1.40	0.62	1.84	1.17; 2.87	0.008
PsoJIA	0.86	0.46; 1.60	0.63	0.79	0.41; 1.51	0.48
UndJIA	0.82	0.42; 1.63	0.58	0.74	0.37; 1.48	0.40
Country						
France	Ref			Ref		
Switzerland	0.80	0.57; 1.12	0.194	1.02	0.72; 1.43	0.92
Patient location area						
Urban	Ref			Ref		
Intermediate	0.97	0.68; 1.38	0.86	0.85	0.60; 1.21	0.37
Rural	1.34	1.00; 1.78	0.047	0.78	0.58; 1.03	0.08
Mother with post-graduate level	1.36	1.02; 1.81	0.036	1.19	0.89; 1.58	0.23
Father with post-graduate level	1.25	0.95; 1.66	0.11	1.01	0.77; 1.34	0.92
At least one parent in a health care profession	0.95	0.65; 1.40	0.80	1.21	0.82; 1.77	0.34
Mother in a health care profession	0.90	0.60; 1.33	0.59	1.40	0.94; 2.09	0.09
Father in a health care profession	1.29	0.64; 2.62	0.48	1.02	0.50; 2.07	0.96

HR = 1 indicates an equal likelihood of a short time to first PR visit in the presence of the variable in question as in its absence. HR > 1 indicates an increased likelihood of a short time to first PR visit. HR < 1 indicates a reduced likelihood of a short time to PR visit

*HR* Hazard ratio, *Ref* Reference, *CI* Confidence interval, *HCP* Healthcare provider, *PR* Pediatric rheumatology, *oJIA* Oligoarticular juvenile idiopathic arthritis, *pJIA* Polyarticular juvenile idiopathic arthritis, *sJIA* Systemic juvenile idiopathic arthritis, *ERA* Enthesitis-related arthritis, *PsoJIA* Psoriatic arthritis, *UndJIA* Undifferentiated juvenile idiopathic arthritis

^a^With a logarithmic transformation

## Discussion

The aim of this study was to highlight factors associated with longer time to access a PRst in France and Switzerland. To our knowledge, this is the first multicenter study in these two countries analyzing access to PR care. Few data are available in France, where studies have covered limited geographical areas and focus mainly on clinical and biological characteristics [16, 27]. No studies had been conducted in Switzerland.

In the present study, the median time to first PR visit was short (2.4 months) compared to other studies [14–25] and close to the British guidelines [13] (children with suspected JIA are to be assessed by a PR team within 10 weeks of symptom onset). However, the data show a broad variability and an excessively long time to access PR centers for many patients (more than 6 months for 27% of patients, and more than 1 year for 14%) while it is known that a late referral can be associated with important damages (as well as articular as ophthalmologic) in most JIA subtypes. This can also impact the quality of the relationship with HCPs involved in the disease management.

As reported previously, sJIA subtype is associated with prompt referral due to eruptive symptoms (fever, rash, deep asthenia, biological inflammatory syndrome) [15, 23, 27]. This is confirmed by our study with a shorter delay between first HCP and first PR visit. In contrast, children with the ERA subtype experienced a significantly longer time to access PR centers. Subtle presentations of JIA with indolent symptoms (e.g. enthesitis without swelling, low biological inflammation, transitional morning stiffness and well-preserved function), as frequently described in ERA subtype, led to a longer time to first PR visit and require specific training and experience in HCPs [15, 16, 20]. In addition, it is possible that children with suspected ERA are less likely to report these symptoms to the attention of family and HCPs. Although this form is generally accompanied by fewer sequelae when treatment is delayed, the negative psychological effects of delay in access to appropriate care, and doubts about diagnosis for both patients and family caregivers should not be overlooked.

The presence of an orthopedist during the referral pathway was significantly associated with a longer time of referral to the PRst. However, in our study, the orthopedist referred most frequently to a PRst (in 75% of the cases, data not shown). The time to get an appointment with an orthopedist and the presence of invasive procedures (e.g., arthroscopies, bone biopsies), that are frequently performed by orthopedist, could explain the overall increase time to the first PR visit [16]. However, studies focusing on care pathways, taking into account the specificities of the organization of health systems in each country, would be necessary.

The care pathway for JIA patients, from first symptoms to appropriate diagnosis and care by a PRst contains two successive intervals: (i) the interval between symptom onset and first assessment by an HCP, mostly depending on patients and family personal and environmental characteristics, and (ii) the referral pathway, namely the interval between first consultation with an HCP and the first PRst assessment, which depends on physicians and healthcare organization performance and effectiveness.

Although previous research found a significant social gradient in health from early childhood [29, 30], the impact of social determinants on children with JIA is poorly understood. In Canada, higher levels of parental education seem to be associated with a shorter time to first PR consultation [15]. Conversely, in the United Kingdom, socio-economic status was not correlated with time to first PR consultation. However, studies found a link between socio-economic factors and different types of pathways: patients with lower socio-economic status were mainly referred to the PRst via the ECP, while patients with higher socio-economic status were mostly referred by a general pediatrician [24]. In the United States, community poverty was associated with delayed time to rheumatology care for patients with pJIA [28]. In our study there was a tendency for shorter symptom duration among children whose mothers had a post-graduate educational level, but this correlation was not statistically significant in multivariable analysis.

In our study, no association was observed between time to access to PR center and rural/intermediate/urban type of living location. This is consistent with 2 previous studies that took place in 2 different areas in France: a densely populated metropolitan area with the highest medical density in the country [27] and a less populated area with lower medical density and encompassing more rural areas [16], from which no differences were observed in access to PR care. Patients in rural area were more likely to experience a shorter time between symptom onset and first visit with an HCP. Although this result may seem surprising (given the low medical density in rural areas), it could be explained by greater pressure on health services due to higher population density in urban areas.

Several studies reported a correlation between socio-economic-level and health literacy [39–41]. The World Health Organization defines health literacy as the ability of individuals to gain access to, use, and understand health information and services in order to maintain good health [42]. Impacts of low health literacy are multiple and adversely affect parents’ ability (especially that of mothers who are generally more involved in their children’s health) to use health information, make health decisions for their child and find their way in the healthcare system (more medication errors, more emergency department use, etc.) [43, 44]. A broader concept of health literacy, such as that measured by the Health Literacy Questionnaire (HLQ), also includes the ability to actively engage with healthcare providers (6^th^ domain of the HLQ) [45]. Patients (or parents in the present case) who are passive in their approach to healthcare (i.e., who do not proactively seek or process information and advice and/or service options), tend to accept information unquestioningly. In contrast, parents who are proactive about their health, and feel in control in relationships with healthcare providers, are able to seek advice from additional healthcare providers when necessary and until they are satisfied. Recent qualitative studies have shown how parents’ determination and self-confidence are important in offsetting the insufficient knowledge of non-specialist physicians. Parental implication is a key factor in referral to appropriate care and it has been shown that parents play a central role at every step in the referral pathway [46]. As reported by Rapley et al., beside the experience and skills of health professionals, “parental persistence” (i.e., persistence in seeking action such as in repeated visits to primary and hospital care to report stubborn symptoms in their child) is crucial for JIA children to access appropriate care [47]. In the present study, although we did not directly measure health literacy, we used the educational level of the mother and father as a proxy for health literacy. We observed a significantly shorter time between first symptoms and access to first HCP when mothers had a post-graduate educational level but there was no association between the mother’s educational level and the time between first HCP visit and access to a PR center. This is consistent with the fact that the time lag before referral was more often due to an HCP’s referral than to a long time before access to the first HCP (2.1 months [1.0; 5.0] *vs.* 0.0 month [0.0; 0.7]). This is in line with Shiff et al., who found that children with rheumatic disease saw an HCP in a median time of 2 weeks after onset of symptoms, whereas the median time to first PR visit was 24 weeks [14]. These results suggest that delays in access to a PR center depend mostly on healthcare organization rather than on patients’ literacy.

This study has some limitations mainly owing to the reduced number of participants in the JIRcohort database whose data were sufficiently complete to be exploitable (only 250 JIA patients for 20 centers). However, characteristics of JIA children included in the study are closely similar to the data reported for Europe (such as frequency of JIA subtypes, age at symptom onset, and female/male ratio) [48]. Moreover, the data observed on the educational attainment level of the parents in our sample are fairly close to the results for the general population in France and Switzerland [49]. The date of the referral letter from the referring physician was not collected, so we can’t evaluate the time lag between referral and assessment by the PRst. Another limitation is the absence of direct measurements of parents’ health literacy. Although there is an overall correlation between educational level and health literacy, some individual domains of health literacy measured by HLQ may be less closely correlated: the ability to engage actively with professionals may depend on other personal characteristics such as self-confidence or psychosocial competencies. Educational attainment level may thus not be an accurate proxy for health literacy.

It would be of interest to supplement these conclusions with data from other sources. However, data on the psychosocial determinants of delay are scant in other databases. Finally, the dates of symptom onset and of HCP’s assessment were declared by the parents, so a memory bias cannot be excluded.

A strength of this study is that it was conducted in a prospective cohort based on 20 centers, which lessens the risk of selection bias observed in monocenter studies.

## Conclusion

In France and Switzerland, the time to first PR visit was most often short compared to other studies, and close to the British recommendations. However, this time was still too long for many patients. We did not observe any social inequities in access to a PRst, but this study does show the need to improve effective pathway and access to a PRst for JIA patients. Qualitative studies are now needed to explore the reasons for this delay between first visit to a practitioner and appropriate referral to a PRst.

## Supplementary Information

**Additional file 1.** Location of the 20 pediatric rheumatology centers (using Google Maps).

## Data Availability

Data are available from the corresponding author on reasonable request.

## Abbreviations

BSPAR: British Society for Paediatric and Adolescent Rheumatology Standards of Care
CI: Confidence interval
ECP: Emergency care practitioner
ERA: Enthesitis-related arthritis
HCP: Healthcare provider
HLQ: Health Literacy Questionnaire
HR: Hazard ratio
ISCED: International Standard Classification of Education
ISCO: International Standard Classification of Occupations
JIRcohort: Juvenile Inflammatory Rheumatism cohort
oJIA: Oligoarticular juvenile idiopathic arthritis
pJIA: Polyarticular juvenile idiopathic arthritis
PsoJIA: Psoriatic arthritis
PR: Pediatric rheumatology
PRst: Pediatric rheumatologist
sJIA: Systemic juvenile idiopathic arthritis
UndJIA: Undifferentiated juvenile idiopathic arthritis
