# Supplementary figures and images for: Individual and environmental determinants associated with longer times to access pediatric rheumatology centers for patients with juvenile idiopathic arthritis, a JIR cohort study
Source: Pediatr Rheumatol Online J. 2023 Mar 14;21:24. doi: 10.1186/s12969-023-00809-8 (PMC10015663; doi:10.1186/s12969-023-00809-8)

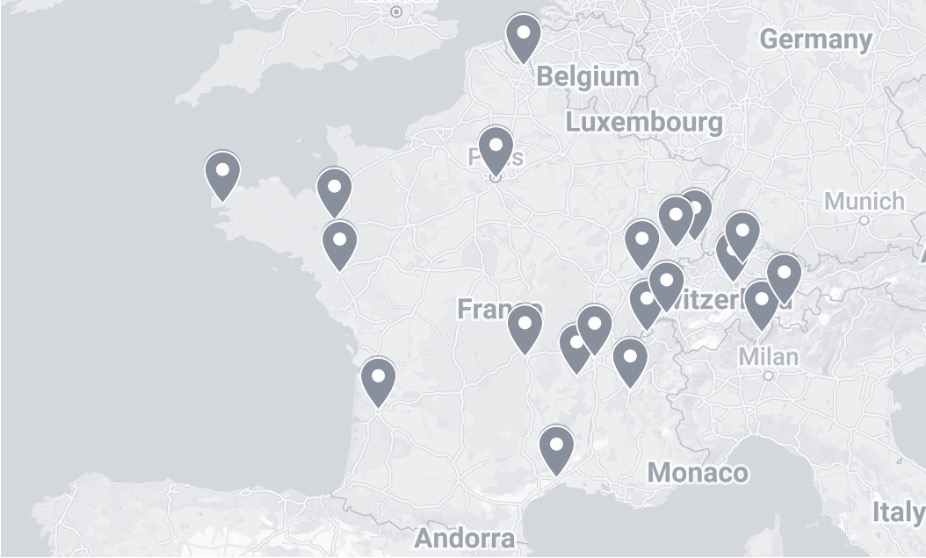


**Additional File 1. Location of the 20 pediatric rheumatology centers (using Google Maps).**

Supplement: Supplementary file 1 — Additional file 1. Location of the 20 pediatric rheumatology centers (using Google Maps). [file 12969_2023_809_MOESM1_ESM.docx]
